# Vaccine-Induced Skewing of T Cell Responses Protects Against Chikungunya Virus Disease

**DOI:** 10.3389/fimmu.2019.02563

**Published:** 2019-10-31

**Authors:** Rebecca M. Broeckel, Nicole Haese, Takeshi Ando, Igor Dmitriev, Craig N. Kreklywich, John Powers, Michael Denton, Patricia Smith, Thomas E. Morrison, Mark Heise, Victor DeFilippis, Ilhem Messaoudi, David T. Curiel, Daniel N. Streblow

**Affiliations:** ^1^Vaccine and Gene Therapy Institute, Oregon Health and Science University, Beaverton, OR, United States; ^2^Department of Radiation Oncology, Washington University, St. Louis, MO, United States; ^3^Department of Immunology and Microbiology, University of Colorado School of Medicine, Aurora, CO, United States; ^4^Department of Genetics, The University of North Carolina at Chapel Hill, Chapel Hill, NC, United States; ^5^Department of Microbiology and Immunology, The University of North Carolina at Chapel Hill, Chapel Hill, NC, United States; ^6^Department of Molecular Biology and Biochemistry, University of California, Irvine, Irvine, CA, United States; ^7^Division of Pathobiology and Immunology, Oregon National Primate Research Center, Beaverton, OR, United States

**Keywords:** Chikungunya virus (CHIKV), vaccine, T cell, pathogenesis, cytokine

## Abstract

Chikungunya virus (CHIKV) infections can cause severe and debilitating joint and muscular pain that can be long lasting. Current CHIKV vaccines under development rely on the generation of neutralizing antibodies for protection; however, the role of T cells in controlling CHIKV infection and disease is still unclear. Using an overlapping peptide library, we identified the CHIKV-specific T cell receptor epitopes recognized in C57BL/6 infected mice at 7 and 14 days post-infection. A fusion protein containing peptides 451, 416, a small region of nsP4, peptide 47, and an HA tag (CHKVf5) was expressed using adenovirus and cytomegalovirus-vectored vaccines. Mice vaccinated with CHKVf5 elicited robust T cell responses to higher levels than normally observed following CHIKV infection, but the vaccine vectors did not elicit neutralizing antibodies. CHKVf5-vaccinated mice had significantly reduced infectious viral load when challenged by intramuscular CHIKV injection. Depletion of both CD4^+^ and CD8^+^ T cells in vaccinated mice rendered them fully susceptible to intramuscular CHIKV challenge. Depletion of CD8^+^ T cells alone reduced vaccine efficacy, albeit to a lesser extent, but depletion of only CD4^+^ T cells did not reverse the protective phenotype. These data demonstrated a protective role for CD8^+^ T cells in CHIKV infection. However, CHKVf5-vaccinated mice that were challenged by footpad inoculation demonstrated equal viral loads and increased footpad swelling at 3 dpi, which we attributed to the presence of CD4 T cell receptor epitopes present in the vaccine. Indeed, vaccination of mice with vectors expressing only CHIKV-specific CD8^+^ T cell epitopes followed by CHIKV challenge in the footpad prevented footpad swelling and reduced proinflammatory cytokine and chemokines associated with disease, indicating that CHIKV-specific CD8^+^ T cells prevent CHIKV disease. These results also indicate that a T cell-biased prophylactic vaccination approach is effective against CHIKV challenge and reduces CHIKV-induced disease in mice.

## Introduction

Chikungunya virus (CHIKV) is a mosquito-transmitted virus that causes fever, rash, and debilitating joint and muscle pain in humans. Though the fever and rash resolve, joint and muscle pain can be long lasting. According to some studies, up to 75% of CHIKV-infected patients experience chronic arthritic and muscle pain for months to years following resolution of the acute disease (1–3). The consequences of chronic joint pain are significant, with patients reporting limited mobility, depression, and decreased quality of life (4). CHIKV can rapidly spread and cause disease in millions of people in a short period of time, as illustrated by recent epidemics in the Indian Ocean region (2004–2011) and the Americas (2013–2015) (5–7). Since no FDA-approved vaccines or antivirals exist for CHIKV, research into prophylactic and therapeutic interventions are highly warranted. The protective role for anti-CHIKV neutralizing antibodies has been well-established in both mouse and non-human primate models (8–11). Potent neutralizing antibodies can provide sterilizing immunity if administered prophylactically or if derived through vaccination. However, after the first few days of infection, neutralizing antibodies may have limited efficacy to clear virus from infected tissues (9), suggesting other immune components, such as T cells, could be involved in viral clearance of persistent joint-localized CHIKV.

CHIKV vaccine candidates under development elicit both humoral and cellular responses to CHIKV antigens. CHIKV vaccines currently being pursued in clinical trials cover a wide range of platforms including virus-like particle (VLP), live-attenuated, viral-vectored, and mRNA-based vaccines (12–16). Vaccine-elicited neutralizing antibodies are the key to protection against CHIKV, but the direct contribution of vaccine-induced T cells in protection against CHIKV is rarely characterized. Two preclinical vaccines, the live-attenuated CHIKV/IRES vaccine and the modified vaccinia virus Ankara (MVA) vectored vaccine, assessed the impact of vaccine-induced T cells (17–20). Both vaccines elicited humoral and cellular responses against CHIKV antigens. However, in the context of vaccine-induced neutralizing antibodies, the roll of cellular immunity appears minimal. In fact, T cell depletion of CHIKV/IRES-vaccinated A129 mice or adoptive transfer of immune CD4^+^ or CD8^+^ T cells did not protect A129 mice from CHIKV (18). However, a modified vaccinia virus ankara (MVA) vaccine vector that expresses E3/E2 was shown to require CD4^+^ T cells for protection since CD4^+^ T cell depletion increased susceptibility to CHIKV challenge (20). While low levels of E2 neutralizing antibodies were identified following vaccination that may have contributed to the protection seen in A129 mice, a role for direct cellular immunity or CD8^+^ T cells was not shown.

CD4^+^ T cells have been implicated as a major contributor to joint inflammation during CHIKV infection of mice. Footpad injection of C57BL/6 mice with CHIKV results in edema, arthritis, and tenosynovitis in the ankle joint, as well as necrosis in the musculoskeletal tissues (21). Infiltrating cells in the ankle include CD4^+^ and CD8^+^ T cells, macrophages, neutrophils, and natural killer cells (21). Mice lacking CD4^+^ T cells have reduced inflammation of the footpad after infection, but viral levels in the blood and ankle are similar to control mice (22). Adoptive transfer of CHIKV-specific CD4^+^ T cells into TCR^−/−^ mice also resulted in increased footpad swelling and joint vascular leakage compared to controls after CHIKV infection, while viral load in the blood remained the same as controls (23). Furthermore, therapeutic administration of mice with CTLA4-Ig after CHIKV infection inhibited T cell recruitment to the ankle and decreased footpad swelling (24). In contrast to CD4^+^ T cells, depletion of CD8^+^ T cells fails to reduce footpad swelling (22). This data supports a pathogenic role for CD4^+^ T cells but the role for CD8^+^ T cells remains unclear. Some evidence suggest T cells promote viral clearance during CHIKV infection. Mice lacking B and T cells (Rag1^−/−^ or Rag2^−/−^) develop more severe persistent infections characterized by chronic viremia and persistence of infectious virus in a number of tissues (9, 25). Passive transfer of neutralizing antibodies into Rag1^−/−^ mice fails to clear CHIKV in tissues of persistently infected animals (9). In addition, mice lacking mature B cells (μMT mice) vaccinated with an inactivated CHIKV vaccine have decreased levels of virus in the serum compared to control-vaccinated mice after CHIKV challenge, although the vaccinated mice also had increased footpad swelling (25). This suggests that vaccine-elicited T cells can provide limited protection, but this was not directly tested. Combined, these data are suggestive of a role of cellular immunity in protection against CHIKV infection.

Antiviral CD8^+^ T cells have been shown to be important for reducing viral loads and disease for other alphavirus infections. Depletion of CD8^+^ T cells in Ross River virus (RRV) infected mice at 7 and 12 dpi increased levels of RRV RNA in the quadriceps at 14 dpi (26). Similarly, infection of CD8α^−/−^ mice with RRV results in increased levels of RRV RNA in the quadriceps at 14 and 21 dpi, but equal levels of virus is detected in the ankle, compared to wild type mice. T cells have also been implicated in protection against Sindbis virus (SINV) infiltration into the CNS (27), and CD4^+^ T cells may protect against Venezuelan Equine Encephalitis virus (VEEV) -induced encephalitis in mice (28).

In the current study, we utilized murine cytomegalovirus (MCMV) and adenovirus (AdV) vaccine vectors as tools to investigate the role for antiviral CD4^+^ and CD8^+^ T cells during CHIKV infection. We profiled T cell epitopes recognized in CHIKV mice using a complete CHIKV (22) overlapping peptide library. Based upon the results from this screen, we generated a CHIKV fusion gene called CHIKVf5 that encodes several peptides that elicited T cell IFNγ responses. The fusion gene was recombined into MCMV and AdV vaccine vectors to elicit CD4^+^ and CD8^+^ T cell responses in mice. After vaccination, we observed earlier joint inflammation in mice challenged in the footpad. However, mice challenged intramuscularly had significant reduction of viral loads in leg muscle tissue. T cell depletion experiments demonstrated that CD8^+^ T cells were essential for protection in the muscle tissue. Mice vaccinated with CD8^+^ T cell epitopes showed decreased CHIKV-induced joint swelling after footpad challenge. This study describes a protective role for CD8^+^ T cells in CHIKV infection and disease.

## Materials and Methods

### Cells

Vero cells, mouse embryonic fibroblasts (NIH/3T3), and 293-IQ cells [HEK293 cells expressing the lac repressor (29)] were propagated at 37°C with 5% CO_2_ in Dulbecco's Modified Eagle Medium (DMEM) supplemented with 5 or 10% Fetal Bovine Serum (FBS) and Penicillin-Streptomycin-L-Glutamine (PSG). *Aedes albopictus* cells (C6/36s) were propagated at 28°C with 5% CO_2_ in DMEM supplemented with 10% FBS and PSG.

### Viruses

CHIKV SL15649 and CHIKV 181/25 was generated from the infectious clones. Briefly, the infectious clone was digested with NotI, and DNA was purified with the QIAquick PCR purification kit (Qiagen) according to the manufacturer's instructions. Viral mRNA was generated with the mMESSAGE mMACHINE SP6 Transcription Kit (ThermoFisher), and the mRNA was purified using the RNeasy Mini Kit (Qiagen). Roughly 3 μg RNA was transfected into Vero cells using Lipofectamine 2000 (ThermoFisher). CHIKV virus stocks were passaged once C6/36 cells for 72 h, and viral stocks were prepared by ultracentrifugation over a 15% sucrose cushion (SW 32 Ti Rotor, 1 h 10 min, 76,755 × g). The virus pellets were resuspended in PBS and aliquots were stored at −80°C. For CHIKV limiting dilution plaque assays, 10-fold serial dilutions of virus stocks or tissue homogenates were plated on Vero cells. The cells were placed on a rocker in an incubator at 37°C with 5% CO_2_ for 2 h, and DMEM containing 0.3% high viscosity carboxymethyl cellulose (CMC) (Sigma) and 0.3% low viscosity CMC (Sigma) was added to the cells. After 2 days, cells were fixed with 3.7% formaldehyde (Fisher), stained with 0.5% methylene blue (Fisher), and dried. Plaques were enumerated under a light microscope.

### MCMV Vectors

The Smith strain MCMV bacterial artificial chromosome (BAC) pSMfr3 (30) was utilized for generating infectious MCMV vaccines. The gene of interest was inserted in-frame onto the C-terminus of the MCMV *IE2* gene so that the insertion is co-expressed with IE2 (31). Generation of the MCMV constructs was performed via a two-step galactokinase/kanamycin (GalK/Kan) cassette insertion and replacement (32, 33). The GalK/Kan cassette was generated by PCR with primers that overlapped *ie2* by 50 bp. The PCR product was electroporated into electrocompetent SW105 cells containing pSMfr3, and bacteria were selected on Kan-containing agarose plates. The fusion gene CHKVf5 was generated by overlapping PCR. A PCR product containing 50 bp homology with *ie2* was generated (F primer: GGTTCTTTCTCTTGACCAGAGACCTGGTGACCGTCAGGAAGAAGATTCAGTGTGCGGTGCATTCGATGAC, R primer: AACCTCTTTATTTATTGATTAAAAACCATGACATACCTCGTGTCCTCTCAGGCGTAGTCGGGCACATC) and electroporated into SW105 cells containing the IE2-GalK/Kan MCMV BAC. Resulting bacteria were selected on 2-deoxy-galactose (DOG) minimal plates, and the presence of the insert was confirmed by PCR and sequencing. Virus was reconstituted by electroporation into NIH/3T3 cells, and passaged five times to eliminate the BAC cassette prior to ultracentrifugation. Constructs were screened by PCR and sequenced to confirm the presence of the insert. MCMVs were titered by plaque assays on NIH/3T3s. Dilutions of virus was plated on NIH/3T3s, and cells were placed in an incubator on a rocker. At 2 hpi, a CMC overlay was added to the cells, and the cells were incubated for 5–7 days, until plaques were formed, prior to fixing and staining with methylene blue.

### Adenovirus Vectors

Replication-defective human Ad5 adenoviruses (del E1, E3) were generated using the AdMax HiIQ system (Microbix). Genes of interest were cloned into the shuttle plasmid pDC316(io) and co-transfected with pBHGloxΔE1,3Cre plasmid into 293 IQ cells to reconstitute virus as previously described (29, 34). Transfections were performed using the PureFection kit (System Biosciences) according to the manufacturer's protocol, and adenovirus plaques were observed after 10–14 days in cell culture. Viruses were passaged four times in 293 IQ cells prior to ultracentrifugation. Constructs were screened by PCR and sequenced to confirm the presence of the insert. Adenovirus titers were calculated using limiting dilution assays on 293 IQs in 96-well plates.

### Mouse Experiments

All mouse experiments were performed at OHSU in ABSL3 laboratories in compliance with OHSU IACUC protocols. The small lab animal unit at OHSU is accredited by the Association for the Accreditation and Assessment of Laboratory Animal Care (AALAC) International. Animals were housed in ventilated racks and monitored daily by veterinary staff. C57BL/6J mice were vaccinated as indicated with MCMV delivered intraperitoneally (10^6^ PFU, i.p.), and/or AdV injected intramuscularly in the thigh (10^8^ PFU, i.m.). Mice were challenged with 10^3^ PFU CHIKV in a 20 μl volume in the footpad (f.p.), or they were challenged (i.m.) with 10^3^ or 10^4^ PFU in a 20 μl volume in the calf muscle. Footpad measurements were taken with calipers. For T cell depletion experiments, mice were administered T cell depleting antibodies diluted in PBS in a 100 μl volume (i.p.). Vaccinated groups were injected with 300 μg anti-CD4 (GK1.5, BioXCell), 300 μg anti-CD8 (2.43, BioXCell), 300 μg Rat IgG2b Isotype Control (LTF-2, BioXCell), or a combination of 300 μg anti-CD4 and 300 μg anti-CD8. T cell depletions were confirmed by flow cytometry. To confirm T cell depletions, splenocytes were stained with fluorophore-conjugated antibodies specific for mouse CD3, CD4, CD8, and CD19. Fluorescent markers were detected on an LSRII instrument (BD Pharminogen) and data was analyzed using FlowJo (TreeStar).

### Western Blot Analysis

NIH/3T3 cells were left uninfected or infected with MCMV CHKVf5 or AdV CHKVf5. Cells were lysed in 1 × Cell Lysis buffer (Cell Signaling) supplemented with 1 mM Phenylmethylsulfonyl fluride (PMSF) (Fisher), scraped, and incubated on ice for 15 min. Lysates were centrifuged (4°C, 10 min, 16,000 × g), and supernatants were transferred to a new tube containing Laemmli buffer. Samples were boiled 10 min and loaded onto 4–12% Bis-Tris SDS-PAGE gels (Thermo/Fisher). Samples were transferred onto Immobilon-P Transfer Membranes (Fisher). Membranes were blocked in 5% milk powder in Tris-buffered saline supplemented with 0.1% Tween 20 (TBST), and membranes were probed with anti-HA antibody (Roche, clone 3F10) at 1:1,000 dilution followed by rabbit anti-Rat HRP-conjugated secondary antibody or anti-GAPDH-HRP (Cell Signaling) for a loading control. Blots were washed with TBST, and the membrane was incubated with chemiluminescent substrate to visualize the bands. The membrane was exposed with X-ray film.

### Neutralization Assays

Blood samples were collected and left at room temperature for 30 min to allow for clotting. Blood was centrifuged (5 min, 3,000 × g) and sera was transferred to a new tube. Sera was heat-inactivated at 56°C for 30 min. Following heat-inactivation, sera was serially diluted in DMEM supplemented with 5% FBS and PSG. Roughly 100 plaque forming units of CHIKV 181/25 were added to serum dilutions, and the complexes were incubated at 37°C for 2 h. Following incubation, complexes were added to Vero cells and placed on a rocker for 2 h at 37°C. CMC-containing media was added to the cells, and cells were placed in a 37°C incubator for 2 days prior to fixing and staining.

### ELISpot Assays

ELISpot assays were performed as previously described (35). Briefly, a single cell splenocyte suspension was prepared by pushing whole spleen through a 70 μm cell strainer and rinsing with 15 ml RMPI complete media (10% FBS, PSG). Cells were pelleted (10 min at 650 × g), and red blood cells were lysed with 1 × Red Blood Cell Lysis Buffer (Bioleged) for 3 min. Splenocytes were replenished with 10 ml RMPI complete and pelleted as before. Cells were resuspended into 5 ml RPMI complete, counted, and samples were normalized to cell count. Splenocytes were added to prewashed Mouse IFNγ ELISpot plates (MabTech) with 1 μl peptide (10 μg/well), 1 μl DMSO, or 1 μl of phorbol 12-myristate 13-acetate/Ionomycin stock as a positive control. Splenocytes were incubated on ELISpot plates for 24–48 h. Plates were washed and incubated with anti-mouse IFNγ biotin antibody for 2 h and streptavidin-ALP antibody for 1 h according to the manufacturer's protocol. Spots were visualized using BCIP/NPT-plus substrate, and plates were rinsed with water and dried prior to counting with an AID ELISPot plate reader.

### Mouse Cytokine Multiplex Assay

A Milliplex MAP Mouse Cytokine Magnetic Bead Panel multiplex assay (Millipore Sigma) was used to quantify the protein levels for 26 cytokines, chemokines, and growth factors in ankle tissues from vaccinated and control mice at 8 days post footpad challenge with CHIKV. Entire ankle samples were homogenized in PBS with 2 mm beads (Propper Manufacturing Co., Inc.) using a Precellys 24 homogenizer (Precellys Bertin Technologies). Cellular debris was pelleted by centrifugation at 5,000 × g for 2 min, and the supernatants were transferred to a new 1.7 ml tube. For the assay, washed antibody-conjugated polystyrene magnetic beads were incubated with a seven-point standard curve or 25 μl of ankle tissue homogenate plus 25 μl of blocking buffer. Samples were incubated at room temperature in the dark for 2 h, washed and then labeled with biotinylated detector antibody for 1 h. Following washing with blocking buffer, the beads were incubated with Phycoerythrin-conjugated streptavidin for 30 min and then washed. Cytokines were quantified using a Luminex 200™ Detection system (Luminex), and the data was analyzed and graphed using GraphPad Prism v6 software.

## Results

### Identification of CHIKV T Cell Epitopes in C57BL/6 Mice

**Figure 1:**
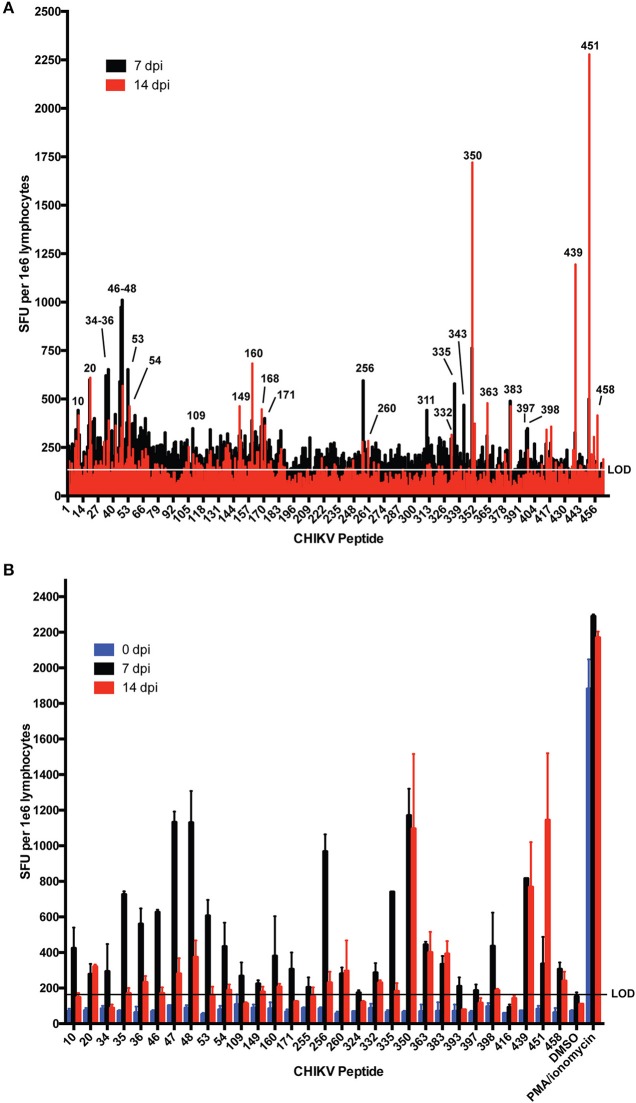
IFNγ responses in C57BL/6 mice at 7 and 14 days post CHIKV infection. Mice were infected with 1,000 PFU CHIKV SL15649 in the footpad. At 7 or 14 dpi, mouse splenocytes were isolated and incubated with individual CHIKV 18-mer peptides overlapping by 10 amino acids and 1e5 cells were incubated on IFNγ ELISpot plates. At 48 h post incubation, ELISpot plates were washed and analyzed for spot formation. **(A)** Splenocytes from three mice were pooled and incubated with each individual CHIKV 18-mer in the CHIKV peptidome. Peptide numbers for reactive samples are indicated. **(B)** IFNγ- eliciting peptides from **(A)** were repeated by ELISpot for two additional animals infected for 7 or 14 days. “LOD” indicates limit of detection.

Few dominant CHIKV T cell epitopes have been experimentally described in C57BL/6 mice (23, 36). To identify T cell epitopes recognized during CHIKV infection of C57BL/6 mice, we screened T cell responses by IFNγ ELISpot using a CHIKV 18mer peptide library with 10 amino acid overlap. For this assay, splenocytes were isolated and pooled from three mice infected with CHIKV for 7 or 14 days (Figure 1A) and cultured on 96-well ELISpot plates for 2 days in the presence of peptides, negative control peptides, or PMA/ionomycin (positive control). Plates were washed and stained for IFNγ. The stained wells were scanned and spots were enumerated. We identified several CHIKV peptides that elicited an IFNγ response that mapped to nsP1, E2, and E1. In addition, we observed a wider breadth of T cell IFNγ responses at 7 dpi (26 peptides recognized) that became more refined at 14 dpi (15 peptides). The screen was repeated (Figure 1B), validating the peptides eliciting the strongest responses as peptides 47, 256, 350, 439, and 451. Peptides 451 (CAVHSMTNAVTIREAEIE) and 350 (DNFNVYKATRPYLAHCPD) were previously shown to elicit T cell responses, providing further validation of our assay (23, 36). Table 1 lists all of the peptides that consistently elicited an IFNγ response above background.

**Table 1:** CHIKV-specific immunoreactive peptides in C57BL/6 mice.

**18-mer peptide sequence**	**Peptide #**	**CHIKV protein**
MSDRKYHCVCPMRSAEDP	10	nsP1
VYAVHAPTSLYHQAIKGV	20	nsP1
HLKGKLSFTCRCDTVVSC	34	nsP1
TCRCDTVVSCEGYVVKRI	35	nsP1
SCEGYVVKRITMSPGLYG	36	nsP1
LNQRIVVNGRTQRNTNTM	46	nsP1
**GRTQRNTNTMKNYLLPVV**	**47**	**nsP1**
**TMKNYLLPVVAQAFSKWA**	**48**	**nsP1**
CCLWAFKKQKTHTVYKRP	53	nsP1
QKTHTVYKRPDTQSIQKV	54	nsP1
RTTNEYNKPIVVDTTGST	109	nsP2
VTWVAPLGVRGADYTYNL	149	nsP2
CVLGRKFRSSRALKPPCV	160	nsP2
PGGVCKAVYKKWPESFKN	171	nsP3
KQHAYHAPSIRSAVPSPF	255	nsP4
**SIRSAVPSPFQNTLQNVL**	**256**	**nsP4**
ELPTLDSAVFNVECFKKF	260	nsP4
GYYNWHHGAVQYSGGRFT	332	Capsid
KPGDSGRPIFDNKGRVVA	335	Capsid
**DNFNVYKATRPYLAHCPD**	**350**	**E2**
GETLTVGFTDSRKISHSC	363	E2
VPKARNPTVTYGKNQVIM	383	E2
MCMCARRRCITPYELTPG	398	E2
**PYSQAPSGFKYWLKERGA**	**439**	**E1**
**CAVHSMTNAVTIREAEIE**	**451**	**E1**
KDHIVNYPASHTTLGVQD	458	E1

*Splenocytes from C57BL/6 mice infected with CHIKV were stimulated with CHIKV-specific 18-mer overlapping peptides, and IFNγ production was measured by ELISpot assays. Consistent immunoreactive peptides are reported, and peptides that elicited the strongest IFNγ responses are in bold lettering*.

### Generation of MCMV and AdV-Vectored Vaccines Directed Against CHIKV

**Figure 2:**
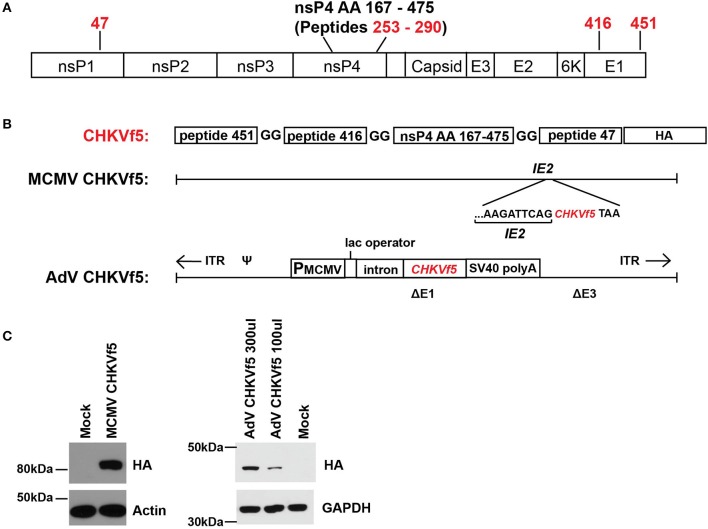
MCMV and AdV vaccine vector expression of the CHKVf5 fusion gene. **(A)** Shown is the genomic position of the CHIKV peptides contained in CHIKVf5. **(B)** The CHIKVf5 fusion gene contains amino acid sequences for peptide 47, 416, and 451 as well as a 308 amino acid portion of nsp4, separated by 2 Gly residues. A C′ terminal HA epitope (8 amino acids) was added for detection purposes. The CHKVf5 fusion gene was inserted into MCMV as a C-terminal fusion with IE2 and into a replication defective adenovirus 5 vector (deleted of E1 and E3). **(C)** Recombinant MCMV and AdV expressing CHKVf5 vectors were tested for HA expression in transduced NIH 3T3 cells. Cell lysates were analyzed at 1 dpi for HA and the loading controls actin or GAPDH.

A fusion polypeptide (CHKVf5) was constructed containing two peptides that elicited strong IFNγ responses (peptide 47 and 451) with a small region of nsP4 (aa 167–475) that was predicted *in silico* to contain several H2-Db restricted T cell epitopes (37). We identified a dominant epitope in peptide 256 in this region of nsP4. Peptide 416 (E1), a peptide that consistently failed to induce IFNγ response by ELISPOT in splenocytes from CHIKV-infected mice, was included as a control peptide (Figure 2A). The CHIKVf5 fusion protein also contained an in-frame C′ terminal HA tag for detection. The fusion construct was generated by overlapping PCR and cloned into shuttle plasmids that allowed recombineering into a MCMV Smith strain bacterial artificial chromosome and recombination into an E1A/E3 deleted AdV genome (Figure 2B). MCMV and AdV were reconstituted, and the insert was confirmed by sequencing. Western blotting for the HA tag in lysates from mouse fibroblasts infected with each of the vaccine vectors confirmed the presence of the fusion protein expression for both constructs (Figure 2C). CHKVf5 was the expected size in AdV CHKVf5-infected cells (about 42 kDa). In the case of MCMV-CHKVf5, CHKVf5 is expressed as an in-frame C-terminal fusion onto IE2, and the IE2-CHKVf5 protein was detected at about 85 kDa.

### Immunogenicity and Efficacy Assessment of MCMV and AdV CHKVf5 Vaccine Vectors

**Figure 3:**
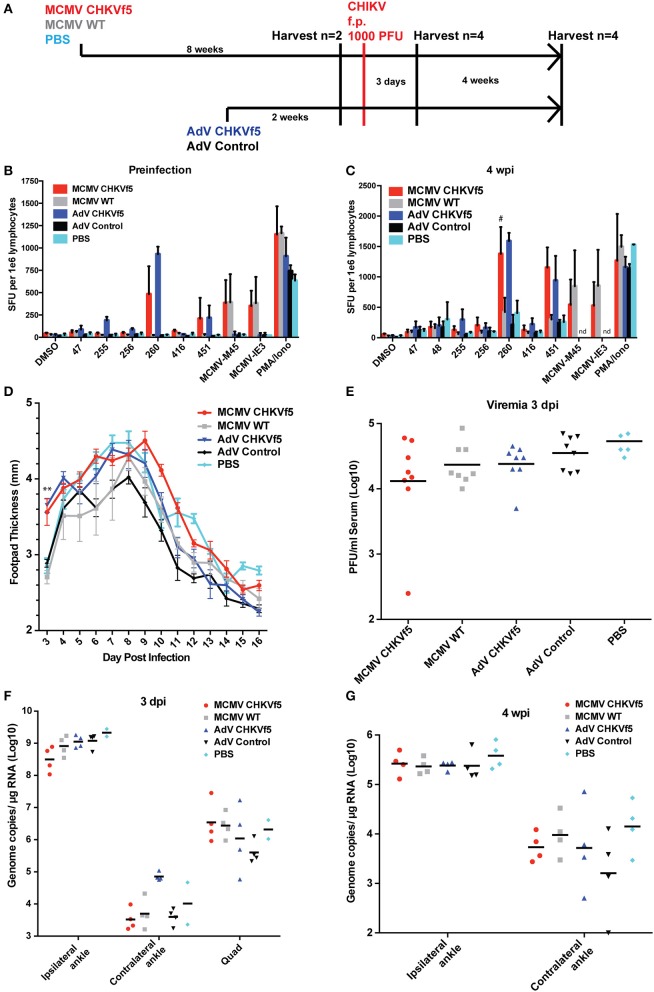
CHKVf5 vaccines did not protect mice against a footpad CHIKV challenge. **(A)** Mice were administered MCMV-CHKVf5, MCMV WT, or PBS i.p. for 8 weeks and then analyzed by ELISpot for the presence of T cell responses or challenged. Separate groups of mice were vaccinated with AdV-CHKVf5 or AdV-Control for 2 weeks. **(B)** Splenocytes from two mice per group were collected and IFNγ ELISpot assays were performed by stimulating the splenocytes with CHIKV peptides incorporated in the CHKVf5 fusion gene. **(C)** IFNγ ELISpot assay performed using splenocytes from mice vaccinated with the indicated vaccine and challenged with CHIKV SL15649 in the footpad (“#” indicates too numerous to count; “nd” indicates not done). **(D)** Footpad thickness was measured using calipers from 3 to 16 dpi. **(E)** At 3 dpi, mice were bled and their serum was titered by limiting dilution plaque assay on confluent monolayers of Vero cells. **(F)** At 3 dpi, viral RNA extracted from ipsilateral and contralateral ankles and ipsilateral quadriceps was quantified by qRT-PCR. **(G)** At 4 wpi, viral RNA was extracted from ipsilateral and contralateral ankles and viral RNA levels were measured by qRT-PCR.

To evaluate vaccine immunogenicity, mice were vaccinated once with either MCMV CHKVf5, wild type MCMV lacking the CHKVf5 insert, or PBS by intraperitoneal injection (*n* = 10/group). Separate groups of 10 mice per group were vaccinated i.m. in the left quadriceps muscle with AdV-CHKVf5 or AdV-control. The vaccine groupings and schedule are depicted in Figure 3A. MCMV vectored vaccines elicit robust CD8^+^ T cell responses by 6–8 weeks post vaccination (31, 38). Adenovirus vaccine-induced CD8^+^ T cell responses peak between 10 and 14 days post vaccination (39). At 8 weeks post MCMV vaccination or 2 weeks post AdV vaccination, splenocytes were isolated from two mice per group for IFNγ ELISpot assays. Splenocytes were incubated with peptides encoded by CHKVf5 (peptides 47, 256, 260, 416, and 451), the immunodominant peptides for the MCMV proteins M45 and IE3, or stimulated with PMA/Ionomycin (Figure 3B). Splenocytes from animals receiving MCMV CHKVf5 and AdV CHKVf5 developed IFNγ responses to stimulation with nsP4 peptide 260 and E1 peptide 451. Splenocytes from mice receiving AdV-CHKVf5 also developed moderate IFNγ responses to stimulation with nsp4 peptide 255. All MCMV-vaccinated mice, but not those receiving AdV, responded to MCMV-specific peptides M45 and IE3.

The remaining animals were challenged with 1,000 PFU CHIKV SL15649 s.c. in the right footpad, and four mice per group were sacrificed at 3 dpi or 28 dpi to measure immunogencity and antiviral efficacy. Twenty-eight days after challenge, IFNγ ELISpots were performed on the splenocytes from all vaccine groups (Figure 3C). While control mice challenged with CHIKV developed IFNγ responses to CHIKV peptides, CHKf5-vaccined mice had a higher frequency of responding cells.

After CHIKV challenge, footpad swelling was monitored from 3 to 16 dpi. Interestingly, the CHKVf5-vaccinated mice had significantly increased footpad swelling at 3 dpi compared to controls (Figure 3D). However, serum viremia and tissue viral loads were not affected by CHKVf5 vaccination (Figures 3E,F). Similarly, there were no significant differences in ankle tissue viral loads at 28 dpi (Figure 3G). These data are largely consistent with studies that showed that T cells do not reduce viral load in the CHIKV footpad infection model (22, 23).

### CHKVf5 Vaccines Induced Protective Responses in Mice Challenged Intramuscularly

**Figure 4:**
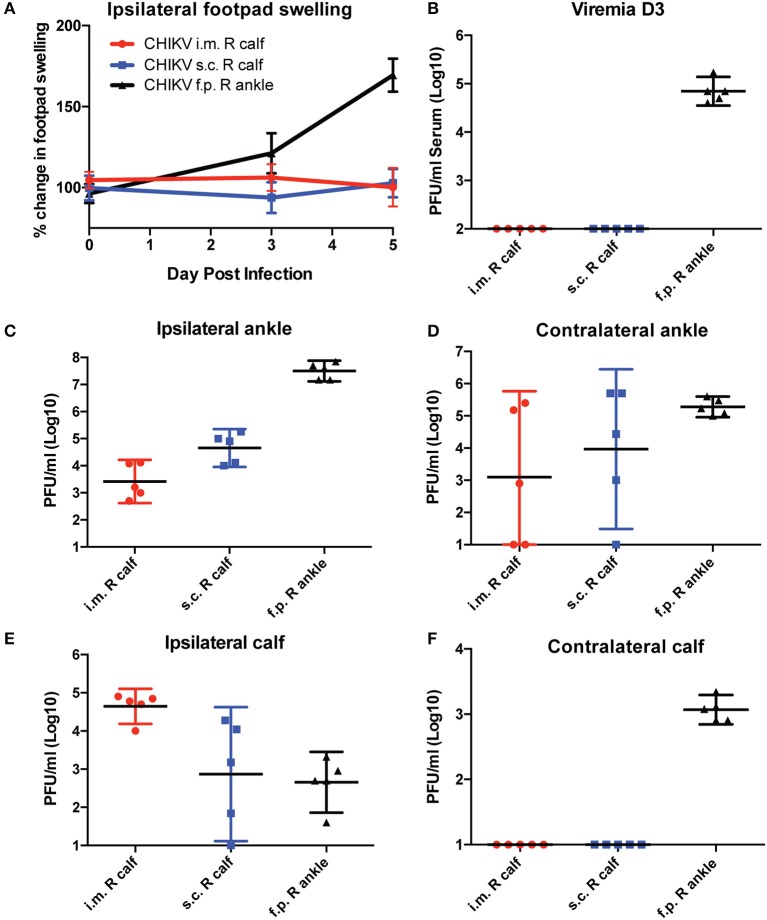
C57BL/6 mice were infected with CHIKV by three different routes, and their footpad swelling and tissue viral distributions are compared. C57BL/6 mice were infected with 1,000 PFU CHIKV SL15649 intramuscularly in the right calf (i.m. R calf), subcutaneously in the skin of the right calf (s.c. right calf), or in the right leg footpad (f.p.). **(A)** Footpad swelling measurements were taken at 0, 3, and 5 dpi using calipers. **(B)** Serum from CHIKV-infected mice was isolated at 3 dpi, and viremia was measured by limiting dilution plaque assay on confluent monolayers of Vero cells. **(C–F)** At 5 dpi, mice were sacrificed and whole tissues were dissected and homogenized in 1 ml cell culture media. Infectious viral levels in the ankle and calf muscle tissue homogenate were measured by limiting dilution plaque assay on confluent monolayers of Vero cells (*n* = 5).

In general, s.c. footpad CHIKV infection quickly replicates to high titers in the ankle, which promotes viral replication and dissemination in a manner that T cells may not be able to control. To test whether the route of infection influences T cell vaccine efficacy, we inoculated mice with 1,000 PFU by three different infection routes (footpad, intramuscular into the calf muscle, or subcutaneous in the calf) in unvaccinated mice and monitored footpad swelling and viral tissue distribution (Figure 4). Only animals inoculated in the footpad developed swelling in the ipsilateral footpad and ankle (Figure 4A). Similarly, only animals inoculated via the footpad route developed measurable viremia at 3 dpi (Figure 4B). Infectious virus was isolated from footpad-infected animals in both ipsilateral and contralateral ankles and calf muscles (Figures 4C–F). Animals inoculated intramuscularly in the right calf had the most consistent and highest levels of virus in the ipsilateral calf at 5 dpi and detectable virus in the ipsilateral ankle, while virus isolation from the contralateral ankle was highly variable. Animals inoculated s.c. in the right calf had detectable virus in the ipsilateral ankle, but levels in the ipsilateral calf and contralateral ankle were variable with no virus detectable in the contralateral calf muscle.

**Figure 5:**
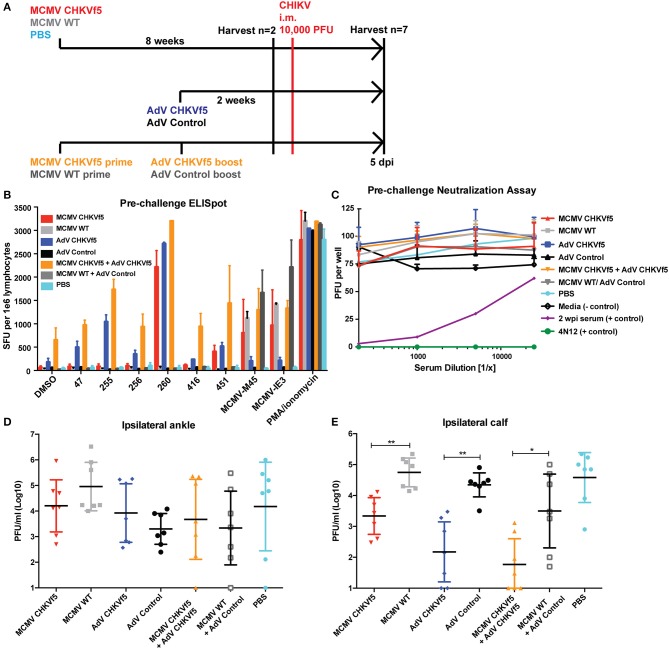
CHKVf5-vaccinated mice do not develop neutralizing antibodies to CHIKV, but their splenocytes produce IFNγ in response to CHIKV peptide stimulation. **(A)** Mice were vaccinated with the indicated vaccine, challenged with CHIKV i.m., and harvested at 5 dpi. **(B)** Prior to CHIKV challenge, IFNγ ELISpot assays were performed on vaccinated mice (*n* = 2), and **(C)** sera from each of the mice were tested for neutralization potential via PRNT assays (*n* = 9). PRNT assay positive controls included convalescent mouse sera from a previously CHIKV infected mouse (shown in purple) as well as a potent neutralizing monoclonal antibody (4N12; shown in green). **(D)** Infectious virus levels detected in the ipsilateral ankle and **(E)** ipsilateral calf. Statistics were reported on log-transformed data using Holm-Sidak's multiple comparison test (*p < 0.05; **p < 0.005; *n* = 7).

Since the i.m. infection group had consistent viral titers in the ipsilateral calf and ankle, we chose to vaccinate mice with CHKVf5 vaccines and challenge i.m. Mice were vaccinated with MCMV and AdV CHKVf5 or control vaccines as described above. A second group of mice received a primary vaccination with MCMV-CHKVf5 or the MCMV control vaccine, and at 8 weeks post prime these animals were boosted with AdV CHKVf5 or AdV control vaccine, respectively (Figure 5A). Prior to challenge with CHIKV, T cell responses were measured in splenocytes from two animals per group by IFNγ ELISpot assays. While splenocytes from AdV-CHKVf5 vaccinated mice produced higher levels of IFNγ expressing T cells in response to CHIKV peptide stimulation compared to the MCMV vaccine platform, T cell response levels were highest for the prime boost approach (Figure 5B). Since two of the peptides included in the CHIKVf5 are derived from E1, we measured neutralizing antibody levels in the serum from mice vaccinated by the different regimens using standard plaque reduction neutralization titer (PRNT) assays. Sera from uninfected, unvaccinated animals was used as a negative control, and immune sera as well as a potent neutralizing monoclonal antibody (4N12) were used as positive controls for PRNT assays (11). Serum from all the CHKVf5 vaccine groups failed to neutralize CHIKV, suggesting that the CHIKVf5 vaccine does not elicit infection neutralizing nor enhancing antibodies (Figure 5C).

Seven mice from each vaccine group were challenged by i.m. calf injection with 10^4^ pfu of CHIKV. At 5 dpi, mice were euthanized and tissues were collected for virological and immunological assessments. Infectious viral titers in the ipsilateral ankle and calf muscle were determined by limiting dilution plaque assays from tissue homogenates (Figures 5D,E). Though there was no statistically significant reduction in virus load in the ipsilateral ankle, there was a significant reduction in infectious virus in the ipsilateral calf from all of the CHIKVf5 vaccine groups (MCMV, AdV, and prime/boost) relative to the appropriate vaccine controls. This finding indicates that the vaccine dramatically reduces viral loads in the calf muscle, but not the ankle tissues.

### CD4^+^ and CD8^+^ T Cells Mediate Protection by the CHKVf5 Vaccine

**Figure 6:**
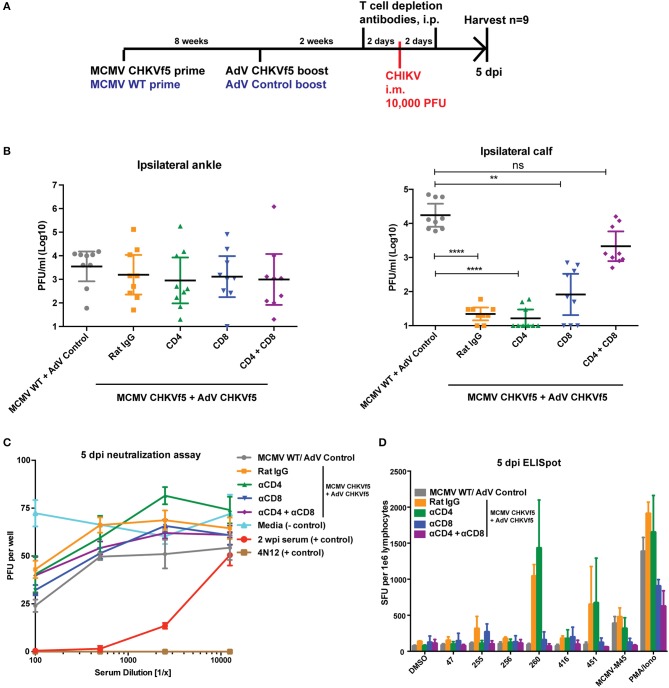
Combined CD8^+^ and CD4^+^ T cell depletion reverses the protective effect of the CHKVf5 vaccine. **(A)** Mice vaccinated with MCMV-CHKVf5 and boosted with AdV-CHKVf5, or control vectors were treated with 300 μg rat isotype control, anti-CD4, anti-CD8, or anti-CD4 plus anti-CD8 depleting antibodies at 2 days prior to challenge. Mice were infected i.m. with 10,000 PFU CHIKV SL15649, and **(B)** ankle and calf tissue viral loads were measured by limiting dilution plaque assays. Statistical analysis was performed on log-transformed data using Dunn's multiple comparison test (**p < 0.005; ****p < 0.0001; *n* = 9). **(C)** Neutralization assays were performed using mouse serum isolated at 5 dpi. **(D)** Splenocytes from vaccinated mice were collected and stimulated with CHIKV peptides on ELISpot plates. The results of the ELISpot assay are reported (*n* = 4).

Since neutralizing antibodies were not detected in the vaccinated animals at the time of challenge, we determined whether protection elicited by the CHIKVf5 vaccine is mediated by the induction of protective T cell responses by T cell depletion experiments in CHIKVf5 prime/boost vaccinated animals. At 14 days post boost (2 days before challenge), animals were infused with depleting antibodies targeting CD4^+^ and/or CD8^+^ T cells (Figure 6A). To confirm effective and specific T cell subset depletion, at 5 dpi splenocytes isolated from three mice per group were collected and analyzed by flow cytometry. As shown in Figure S1, depletion of specific T cell subpopulations (CD4 and/or CD8) were appropriately depleted to levels of >95%. At 5 days post challenge, CHKVf5-vaccinated mice that were untreated or those receiving control rat IgG or CD4^+^ T cell depleting antibodies had significantly reduced levels of infectious CHIKV present in the calf muscle relative to animals that received the control vaccine. These groups were not significantly different from each other suggesting that CD4^+^ T cells alone do not play a major role in CHIKVf5-mediated protection. However, depletion of CD8^+^ T cells increased levels of infectious CHIKV present in the calf, which was enhanced in mice depleted for both CD4^+^ and CD8^+^ T cells. This finding indicates that while CD8^+^ T cells are required for vaccine-mediated protection against CHIKV i.m. challenge, CD4^+^ and CD8^+^ T cells cooperate to mediate protection in the calf muscle.

As expected, similar viral levels were detected in the ankles for all groups (Figure 6B). Data in Figure 6C demonstrate that CHIKV neutralizing antibodies were not present in the vaccinated mice prior to CHIKV challenge. In addition, splenocytes from the various groups were analyzed by peptide ELISpot assay, shown in Figure 6D. These data show that *in vivo* depletion of CD4^+^ T cells eliminated IFNγ responses for peptide 255, but responses for peptides 260 and 451 were only blocked by depletion of CD8^+^ T cells. These data indicate that maximum muscle tissue protective immunity elicited by the CHKVf5 vaccine is mediated by both CD4^+^ and CD8^+^ T cells that act in concert to reduce viral loads in the muscle tissue.

### CD8-Specific AdV 260 and AdV 451 Reduce CHIKV Disease

**Figure 7:**
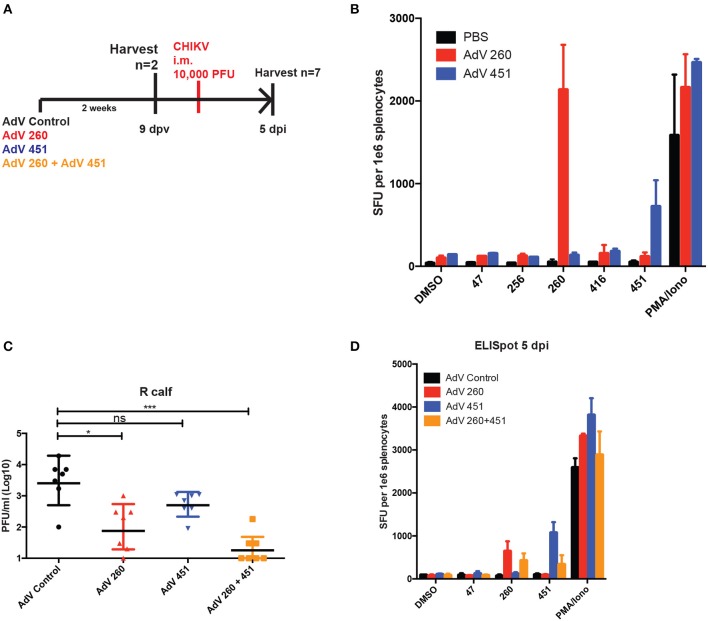
AdV-260 and AdV-451 vaccination elicits T cells responses and protects mice from CHIKV in ipsilateral calf. **(A)** Mice were vaccinated with AdV-260 and/or AdV-451 or PBS (i.m.). Mice were infected i.m. with 10,000 PFU CHIKV SL15649. **(B)** Prior to CHIKV challenge, IFNγ ELISpot assays were performed on a subset of vaccinated mice (*n* = 2). **(C)** Virus was measured in the ipsilateral footpad by qRT-PCR at 5 dpi. Statistical analysis was performed on log-transformed data using Dunnett's multiple comparison test (ns, not significant; *p < 0.05; ***p < 0.0005; *n* = 7). **(D)** At 5 dpi, IFNγ ELISpot assays were performed on splentocytes from a subset of vaccinated mice (*n* = 3).

Since CD8^+^ T cells were essential for the protective effects of the CHKVf5 vaccine, we generated adenoviruses individually expressing the CD8-specific CHIKV peptides 260 and 451. CHIKV-specific T cell responses in mice vaccinated with AdV-Control, AdV-260, or AdV-451, demonstrated appropriate IFNγ responses against peptides 260 and 451 (Figures 7A,B). An additional cohort of these vaccinated mice were challenged with CHIKV i.m. AdV 260 vaccination alone resulted in significantly reduced virus in the ipsilateral calf, but AdV-451 vaccination, while trending lower than controls, was not statistically significantly different than mice vaccinated with a control AdV (Figure 7C). Mice vaccinated with both AdV-260 and AdV-451 had a highly significant decrease in infectious virus compared to controls. The vaccinated and challenged mice also showed vaccine-specific T cell responses after 260 and 451 peptide stimulation (Figure 7D). Therefore, the CD8^+^ T cells elicited after vaccination with adenovirus-delivered peptides 260 and 451 are protective against i.m. CHIKV challenge.

**Figure 8:**
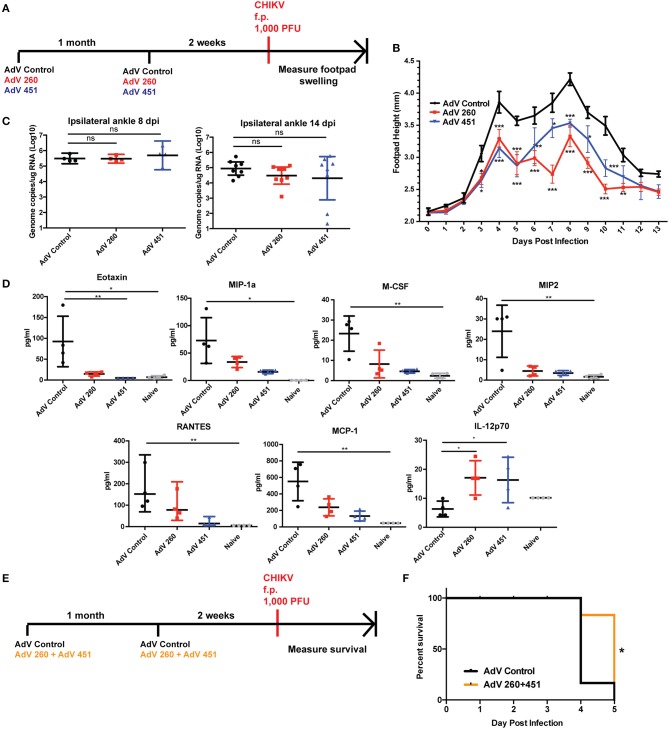
AdV-260 and AdV-451 vaccination reduces inflammatory cytokine production, protects mice from CHIKV-induced footpad swelling, and increases survival in *Ifnar*^−/−^ mice. **(A)** Mice were vaccinated and boosted with AdV-260, AdV-451, or AdV-Control (i.m.) followed by 1,000 PFU CHIKV challenge in the right footpad. **(B)** Footpad swelling was measured for 13 days after CHIKV footpad challenge. Statistics were performed using Dunnett's multiple comparison test (*p < 0.05; **p < 0.005; ***p < 0.0005; *n* = 7) **(C)** Virus was measured in the ipsilateral footpad by qRT-PCR at 8 and 14 dpi. Statistical analysis was performed on log-transformed data using Dunnett's multiple comparison test. **(D)** Ipsilateral ankle cytokine and chemokines were measured at 4 dpi using a 26-plex mouse cytokine and chemokine kit. Statistical analysis was performed using Kruskal-Wallis test followed by Dunn's multiple comparison post-test (*p < 0.05; **p < 0.005; *n* = 4). **(E)** *Ifnar*^−/−^ mice were vaccinated and boosted with either AdV-Control or both AdV-260 and AdV-451. Mice were challenged with 1,000 PFU CHIKV in the footpad. **(F)** Survival of *Ifnar*^−/−^ mice vaccinated with AdV-Control or AdV-260 and AdV-451 and challenged with CHIKV. Statistics were performed using Log-rank test (*p < 0.05; *n* = 10).

We next tested efficacy of disease protection for the AdV-260 and AdV-451 vaccines using the f.p. CHIKV challenge model as outlined in Figure 8A. Footpad swelling at both of the biphasic peaks occurring at day 4 and 8 post infection was significantly reduced in mice vaccinated and boosted with AdV-260 or AdV-451 compared to mice receiving the control AdV (Figure 8B), despite having no statistically significant effect on CHIKV viral RNA loads in the ipsilateral ankles at 8 and 14 dpi (Figure 8C). Due to this response, we hypothesized that pre-existing antiviral CD8^+^ T cells may be associated with differential expression of proinflammatory cytokines and chemokines in the CHIKV-infected ankles. We therefore performed a multiplex cytokine assay using ipsilateral ankle homogenates harvested at 4 dpi from mice vaccinated with AdV control, AdV 260, and AdV 451. From this we observed significantly higher levels of Eotaxin in challenged AdV Control mice relative to Adv 260, Adv 451, and naïve animals that are unchallenged and unvaccinated (Figure 8D). Levels of MIP-1α, M-CSF, MIP2, MCP-1, and RANTES were also elevated in AdV control relative to naïve but not AdV 260 or AdV 451 groups. Interestingly, IL-12p70 was significantly increased in the AdV 260 and AdV 451 groups compared to AdV Control, suggesting a positive role for IL-12p70 in controlling the CHIKV-induced inflammation after vaccination.

We next tested whether vaccination with AdV-260 and AdV-451 could improve survival in a lethal CHIKV mouse model. Mice lacking the type I IFN receptor (*Ifnar1*^−/−^*)* are extremely susceptible to CHIKV and succumb to virus infection 3-4 dpi (40), and represent a highly stringent survival model. Mice receiving AdV-260 and AdV-451 exhibited a 1-day advantage in survival over the AdV-Control group (Figures 8E,F). Therefore, the CD8^+^ T cell vaccine could protect immunocompetent mice from footpad swelling after CHIKV f.p. challenge, and the vaccine provided significant survival advantage in *Ifnar1*^−/−^ mice.

## Discussion

While the development of neutralizing antibodies is a clear immunological correlate of protection for CHIKV, it was unknown what role T cells play in protection against CHIKV infection and disease. In the current study, we mapped the T cell responses to CHIKV in C57BL/6 mice using an overlapping CHIKV peptide library in order to identify potential CHIKV targets for development of a T cell vaccine. During this T cell profiling experiment, we identified 26 unique peptide sequences that elicited significant IFNγ responses in splenocytes obtained from CHIKV-infected C57BL/6 mice. Based upon this information, we constructed a fusion polyprotein that contained the amino acid sequence for a number of these positive CD4^+^ and CD8^+^ restricted peptides. This CHKVf5 fusion gene was inserted into two different T cell promoting vaccine platforms including MCMV and AdV. Vaccinated mice developed robust T cell responses directed against the transgene construct that were amplified following challenge with CHIKV. Vaccination prior to footpad inoculation resulted in a dramatic increase in footpad swelling at 3 dpi. We attribute this to preformed anti-CHIKV CD4^+^ T cells. While the CHKVf5 vaccine did not protect against high ankle viral loads following challenge, it did significantly reduce viral loads in the calf muscle when i.m. challenged with CHIKV.

Two crucial findings indicate that the CHKVf5 vaccine elicits protection through T cells. First, depletion of CD4^+^ and CD8^+^ T cells at 2 days before challenge from vaccinated mice negated the protective efficacy of the vaccine against i.m. challenge by restoring viral loads to levels observed in vaccine controls and non-vaccinated mice. It appears that both T cell subtypes contribute to immune efficacy since depletion of either CD4^+^ or CD8^+^ T cells failed to fully restore CHIKV tissue load to control levels. However, depletion of CD8^+^ T cells significantly reduced the ability of mice to control virus indicating a dominant role for these cells. We presume that CD4^+^ T cell help is required for full efficacy but these cells specifically enhance CHIKV inflammatory joint disease. Second, the CHKVf5 vaccine does not contain any known CHIKV neutralizing domains. As such, we did not detect neutralizing antibodies against CHIKV in any of the vaccinated mice, and the generation of antibody responses against CHIKV following challenge developed with normal kinetics and amplitude when compared to controls indicating that there was no amnestic-type response. Together, these data demonstrate that the CHKVf5 vaccine constructs elicit robust T cell responses that protected against CHIKV in muscle tissues. Finally, we showed that vaccination with adenoviruses containing CD8^+^ T cell epitopes did not decrease joint tissue viral loads after footpad challenge, but it reduced footpad swelling and inflammation following CHIKV challenge. In addition, the CD8^+^ T cell vaccine provided enhanced survival for *Ifnar1*^−/−^ mice.

T cell responses directed against CHIKV have been reported in humans during both the acute and chronic phases of infection. During the acute phase, there is a mobilization and amplification of activated CD8^+^ T cells, followed by CD4^+^ T cells (41). Following the acute phase, patients who recovered from CHIKV and patients with chronic CHIKV-induced arthritis both had roughly equal frequencies of CHIKV-specific IFNγ-producing T cells (42). There is also evidence that T cells can enter CHIKV infected joint tissues in humans. In a synovial biopsy of a patient with chronic CHIKV-induced arthritis, activated (HLA-DR^+^) CD4^+^ T cells were identified as a major cellular infiltrate, but oddly, CD8^+^ T cells were rarely found (1). CHIKV RNA and antigen have been detected in joint synovial biopsies and muscle tissue (1, 43), which is suggestive of viral persistence in the joints and muscle. Though it is not known whether T cells protect against CHIKV in humans, our data would suggest that the presence of effective antiviral CD8^+^ T cells may promote viral clearance in the muscle tissue and control joint inflammation.

We showed that vaccinated animals had a significant reduction of CHIKV titers in the calf muscle. The tissue-specific protection is reminiscent of the study with RRV, where protection by CD8^+^ T cells was observed in the muscle tissue during RRV infection (26), but CD8^+^ T cells failed to reduce viral loads in the ankle tissues. Similarly, we found that CHKVf5-elicited T cells and peptide 260 and 451-elicited T cells were unable to significantly reduce viral burden in the ankles. Similar tissue-type targeting occurs following SINV infection, where T cells are important for viral clearance in the brain and spinal cord (27). In addition, CD4^+^ T cells were important for protection mediated by a live-attenuated vaccine for VEEV (28). Together these studies demonstrate the importance of T cells in alphavirus clearance from infected tissues.

Interestingly, we found that T cell responses elicited by CHIKVf5 were more frequent and directed against different epitopes when compared to those observed following CHIKV infection. For example, splenocytes from CHIKV infected mice had high frequencies of IFNγ responses to peptides 47-48, 256, 350, 439, and 451. While T cell responses against peptides 350 and 439 were robust, the CHKVf5 did not contain these because it was designed prior to us determining whether these were CD4^−^ or CD8^−^ specific. Future T cell vaccine constructs would most likely add these epitopes as they may impact protection. Since peptides 47, 256, and 451 were included in the CHKVf5 fusion gene, we expected that vaccinated mice would primarily induce responses to those epitopes. However, peptide 260 and 451 elicited the highest frequency of IFNγ^+^ T cells in splenocytes from CHKVf5 vaccinated mice indicating that vaccine-induced responses were skewed toward infection-associated, non-dominant epitopes. Thus, one could argue that the protection elicited by the CHKVf5 vaccine may be due to this skewing of the immune response and higher frequencies of responding T cells. Similarly, while both MCMV- and AdV-CHKVf5 vaccines elicited strong T cell responses, the breadth of the responses was vaccine vector-dependent. The AdV-CHKVf5 vaccine directed responses against peptides 47, 255, 256, 260, and 451, while MCMV-CHKVf5 mainly directed T cells against 260 and 451. Prime-boost with both vectors increased the frequency and breadth of responses. Adenovirus vectors elicit potent effector and central memory CD8^+^ T cells (44) whereas MCMV vectors, in general, elicit long-lasting effector memory T cells to vaccine antigens fused to the C-terminus of the IE2 gene (31, 38, 45). Together, the MCMV prime/AdV boost may offer advantages in induction of a diverse T cell memory response to CHIKV epitopes resulting in a more protective phenotype.

Neutralizing antibodies are an important correlate of protection for CHIKV, and it would be essential for a prophylactic CHIKV vaccine to induce neutralizing antibody responses to protect from CHIKV acquisition. The data presented here show that a CD8-directed T cell component in a prophylactic vaccine would be beneficial. This type of vaccine could also be used to prophylactically boost existing immunity and/or skew the responses toward additional epitopes for increased protection against CHIKV acquisition. This approach could be effective for eliminating CHIKV-induced myalgia and joint swelling in infected patients. Therefore, future studies will examine the effects of therapeutic T cell vaccines against CHIKV persistence and disease.

## Data Availability Statement

This manuscript contains previously unpublished data and materials that are available upon request.

## Ethics Statement

The animal study was reviewed and approved by Oregon National Primate Research Center (ONPRC) Institutional Animal Care and Use Committee.

## Author Contributions

RB, NH, TA, ID, CK, JP, MD, PS, IM, and DS performed the experiments. RB, TM, MH, VD, DC, and DS wrote and edited the manuscript. All authors contributed to discussions about the results and their impact.

### Conflict of Interest

The authors declare that the research was conducted in the absence of any commercial or financial relationships that could be construed as a potential conflict of interest.

## Supplementary Material

Figure S1 CD4^+^ and CD8^+^ T cell depletions were confirmed in splenocytes of infected mice. Levels of CD4^+^ and CD8^+^ T cells were measured in splenocytes from mice that received Rat isotype IgG, anti-CD4, or anti-CD8 antibodies by flow cytometry.

## Abbreviations

CHIKV: Chikungunya virus
RRV: Ross River virus
SINV: Sindbis virus
VEEV: Venezquelan equine encephalitis virus
MCMV: mouse cytomegalovirus
AdV: Adenovirus 5
ELISA: enzyme-linked immunosorbent assay
ELISpot: enzyme-linked immune adsorben spot
MIP-1α: macrophage inflammatory protein-1alpha
MIP-2: macrophage inflammatory protein-2
MCP-1: monocyte chemoattractant protein-1
M-CSF: macrophage colony stimulating factor
IL-12: interleukin 12
RANTES: regulated upon activation, normal T cell expressed, and secreted
CNS: central nervous system
CTLA4: cytotoxic T-lymphocyte-associated protein 4
MHC: major histocompatibility antigen
HLA-DR: human leukocyte antigen DR isotype.
