# Supplementary figures and images for: Vaccine-Induced Skewing of T Cell Responses Protects Against Chikungunya Virus Disease
Source: Front Immunol. 2019 Oct 31;10:2563. doi: 10.3389/fimmu.2019.02563 (PMC6834551; doi:10.3389/fimmu.2019.02563)

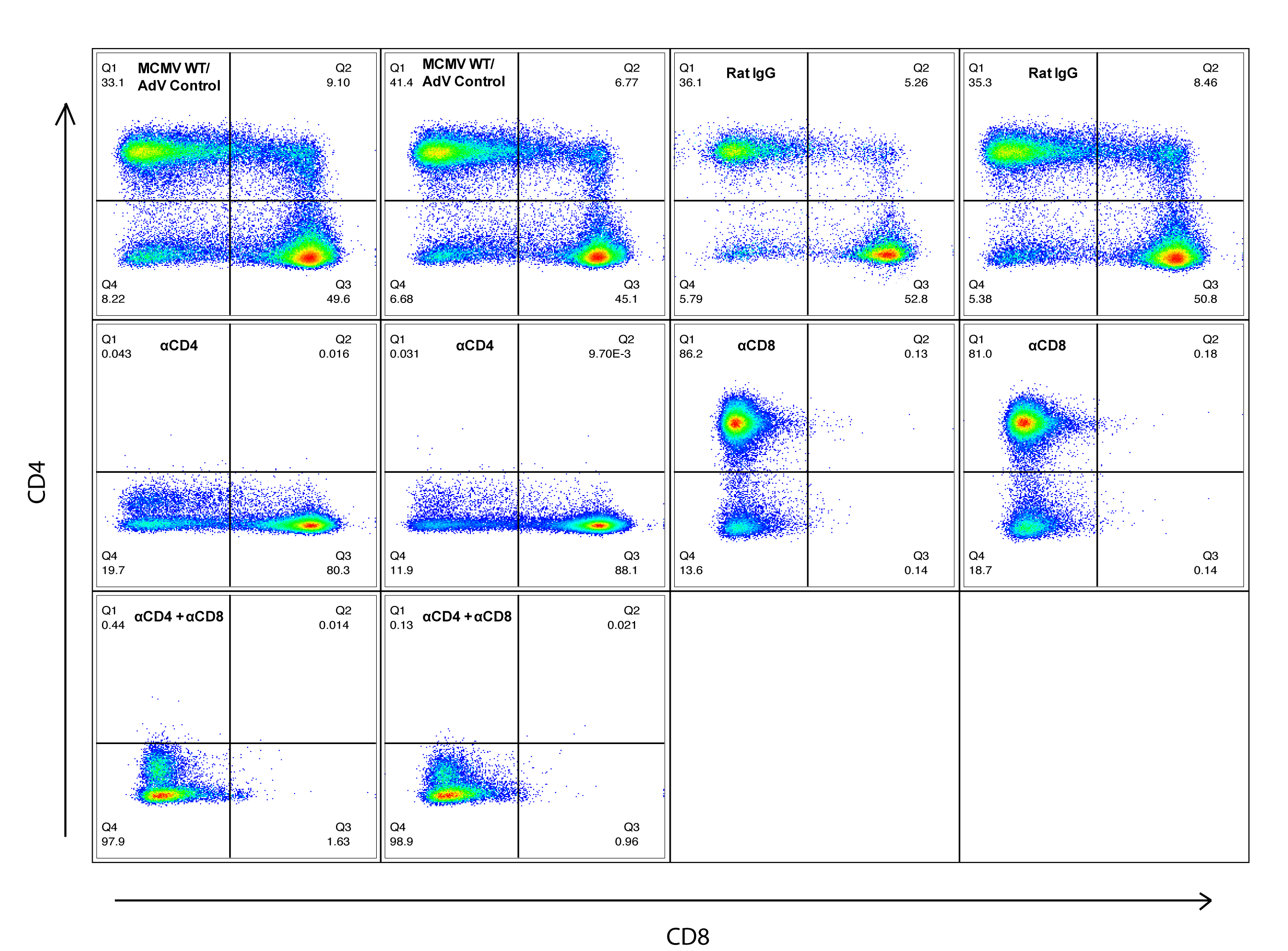

Supplement: Figure S1 — CD4+ and CD8+ T cell depletions were confirmed in splenocytes of infected mice. Levels of CD4+ and CD8+ T cells were measured in splenocytes from mice that received Rat isotype IgG, anti-CD4, or anti-CD8 antibodies by flow cytometry. [file Image_1.TIF]
